# Supplementary material for: Epidemiology and preclinical management of dog bites among humans in Wakiso and Kampala districts, Uganda: Implications for prevention of dog bites and rabies
Source: PLoS One. 2020 Sep 21;15(9):e0239090. doi: 10.1371/journal.pone.0239090 (PMC7505423; doi:10.1371/journal.pone.0239090)
Supplement: S1 Table — The factors were organized into host / patient factors, including socio-demographics and those that influence the vulnerability of people to dog bites. Some factors on biting dogs were also studied to give a clear indication of how they influence the dogs to bite as well as the practices of the victims after the bite. Factors on the circumstances of the particular dog bite event were categorized into pre-bite, during the bite and post-bite factors. The intention of this was to study why the event happened and the wound management practices thereafter. The measures indicate how the variable was recorded and / or categorized. (DOCX) [file pone.0239090.s001.docx]

**S1 Table: Variables that were studied, indicating their categorization and measures**

The factors were organized into host / patient factors, including socio-demographics and those that influence the vulnerability of people to dog bites. Some factors on biting dogs were also studied to give a clear indication of how they influence the dogs to bite as well as the practices of the victims after the bite. Factors on the circumstances of the particular dog bite event were categorized into pre-bite, during the bite and post-bite factors. The intention of this was to study why the event happened and the wound management practices thereafter. The measures indicate how the variable was recorded and / or categorized.

| ***Category of variable*** | ***Variable*** | ***Measure*** |
| --- | --- | --- |
| Sociodemographic factors | Age | lived years from date of birth, then categorized into ≤15 and ˃15 years. |
|  | Sex | male / female |
|  | Religion | Christian / non-Christian |
|  | Highest education level attained | no formal education / primary / secondary and above) |
|  | Marital status | in union or not in union |
|  | Household size | number of people participant stays with at home |
| Patient factors | Presence of teenagers in home | yes / no |
|  | Dog ownership | yes / no |
|  | Ever been bitten by dog before current episode | yes / no |
|  | Vaccination against rabies | yes / no |
|  | Socioeconomic status | determined through the use of a principal components analysis based on household possession of these items (yes/no): radio, television, cell-phone, bicycle, motorcycle, motor vehicle, a piece of land, large farm animals (like cattle, goats and sheep), small farm animals like poultry, a manufactured bed and nature of walls of the house (no bricks / unburnt bricks / burnt bricks with mud / burnt bricks ⁄ stones with cement). |
|  | access to dog bite information | yes / no |
|  | source of information on dog bite | friends / books /school / family / other and specify |
|  | Patient thins bite was intentional | yes / no |
|  | if there is anybody to blame for the bite | yes / no, if yes, mention who it is. |
| Factors about the dog |  |  |
|  | Sex | male / female / unknown |
|  | Sickly appearance | yes / no / don’t know |
|  | Exhibiting fear of people | yes / no |
|  | Vaccination status | yes / no / don’t know |
|  | Spay or castration status | yes / no / don’t know |
|  | Biting another person successively | yes / no / do not know |
| **Circumstances of the bite** |  |  |
| Before the bite | Time of bite | morning / evening / night |
|  | Raining | yes / no |
|  | Moonlight | yes / no |
|  | Bitten by own | yes / no |
|  | Bitten on property of dog owner | yes /no |
|  | Owner around during bite | yes /no |
|  | Size of dog | small / medium / large / very large |
|  | Known breed | yes / no |
|  | Ever known this dog | yes /no |
|  | In company of another person | yes /no |
|  | Activity before bite | walking / seated / chasing it away / feeding it / other |
|  | Dog activity before bite | describe |
|  | Demeanor or mood of dog | describe |
| During the bite |  |  |
|  | Patient approached the dog | yes /no |
|  | Dog movement | stationary / mobile |
|  | Purpose of interaction with dog | describe |
|  | Patient tried to fend off dog | yes /no |
|  | Location of bite on body | leg / hand / abdomen / other and specify |
|  | Number of bites | one / two / three or more |
|  | Patient’s perception of depth of wounds | scratch / shallow / very deep |
| After the bite |  |  |
|  | Action on dog post bite | chased away, killed, nothing, ran away, other and specify |
|  | Action on carcass if killed | decapitated / buried / left to rot / don’t know / other and specify |
|  | Awareness of head examination | yes / no |
