# Supplementary material for: Epidemiology and preclinical management of dog bites among humans in Wakiso and Kampala districts, Uganda: Implications for prevention of dog bites and rabies
Source: PLoS One. 2020 Sep 21;15(9):e0239090. doi: 10.1371/journal.pone.0239090 (PMC7505423; doi:10.1371/journal.pone.0239090)
Supplement: S2 Table — The characteristics were reported by the dog bite victims or their caretakers if they knew the details of the biting dog. The frequencies of such characteristics are presented by the specific districts of bite event and the differences in the distribution of characteristics are indicated by the corresponding p-value. Notably, all study participants were residents of the district where the bite event happened. (DOCX) [file pone.0239090.s002.docx]

**S2 Table: Characteristics of biting dogs as reported by the 376 study participants from Wakiso and Kampala districts, Uganda**

The characteristics were reported by the dog bite victims or their caretakers if they knew the details of the biting dog. The frequencies of such characteristics are presented by the specific district where the bite events happened, and the differences in the distribution of characteristics are indicated by the corresponding *p-value*. Notably, all study participants were residents of the district where the bite event happened.

| **Characteristics** | **Frequency N=276** | **Wakiso** | **Kampala** | **p-value** |
| --- | --- | --- | --- | --- |
| Sex |  |  |  |  |
| Male | 133 (35.4) | 73 (38.4) | 60 (32.3) |  |
| Female | 73 (19.4) | 33 (17.4) | 40 (21.5) |  |
| Don’t know | 170 (45.2) | 84 (44.2) | 86 (46.2) | 0.382 |
| Rabies vaccination status |  |  |  |  |
| No | 50 (13.3) | 30 (15.8) | 20 (10.8) |  |
| Yes | 41 (10.9) | 24 (12.6) | 17 (9.1) |  |
| Don’t know | 285 (75.8) | 136 (71.6) | 149 (80.1) | 0.154 |
| Dog looked sick |  |  |  |  |
| No | 250 (66.5) | 133 (70.0) | 117 (62.9) |  |
| Yes | 73 (19.4) | 38 (20.0) | 35 (18.8) |  |
| Don’t know | 53 (14.1) | 19 (10.0) | 34 (18.3) | 0.069 |
| Exhibited fear of people |  |  |  |  |
| No | 253 (67.3) | 129 (67.9) | 124 (66.7) |  |
| Yes | 102 (27.1) | 54 (28.4) | 48 (25.8) |  |
| Don’t know | 21 (5.6) | 7 (3.7) | 14 (7.5) | 0.254 |
| Breed of dog |  |  |  |  |
| Local | 222 (59.0) | 103 (54.2) | 119 (64.0) |  |
| Crossbreed and exotic | 48 (12.8) | 27 (14.2) | 21 (11.3) |  |
| Don’t know | 106 (28.2) | 60 (31.6) | 46 (24.7) | 0.156 |
| Bitten someone before |  |  |  |  |
| No | 73 (19.4) | 38 (20.0) | 35 (18.8) |  |
| Yes | 77 (20.5) | 49 (25.8) | 28 (15.1) |  |
| Don’t know | 226 (60.1) | 103 (54.2) | 123 (66.1) | 0.023* |
| Bitten someone after |  |  |  |  |
| No | 104 (27.7) | 59 (31.1) | 45 (24.2) |  |
| Yes | 76 (20.2) | 41 (21.6) | 35 (18.8) |  |
| Don’t know | 196 (52.1) | 90 (47.4) | 106 (57.0) | 0.163 |
| Dog size |  |  |  |  |
| Small | 69 (18.4) | 32 (16.8) | 37 (19.9) |  |
| Medium | 167 (44.4) | 86 (45.3) | 81 (43.6) |  |
| Large | 140 (37.2) | 72 (37.9) | 68 (36.6) | 0.747 |
| Mood interpreted |  |  |  |  |
| No | 193 (51.3) | 99 (52.1) | 94 (50.5) |  |
| Yes | 183 (48.7) | 91 (47.9) | 92 (49.5) | 0.761 |
| Dog owner known |  |  |  |  |
| No | 201 (53.5) | 93 (48.9) | 108 (58.1) |  |
| Yes | 175 (46.5) | 97 (51.1) | 78 (41.9) | 0.076 |
