# Supplementary material for: Epidemiology and preclinical management of dog bites among humans in Wakiso and Kampala districts, Uganda: Implications for prevention of dog bites and rabies
Source: PLoS One. 2020 Sep 21;15(9):e0239090. doi: 10.1371/journal.pone.0239090 (PMC7505423; doi:10.1371/journal.pone.0239090)
Supplement: S3 Table — Five themes were synthesized out of the in-depth interviews to explain the circumstances in which the bite events happened. Immediate actions taken by bite victims were categorized into two. For those who applied different substances to the bite wounds, the reasons for their choice and actions were recorded into five categories. The same was done to explain why victims went to herbalists, healthcare facilities, or had a simultaneous resort. (DOCX) [file pone.0239090.s003.docx]

**S3 Table: Summary of circumstances of the dog bites, immediate actions taken by victims and reasons for different applications and health seeking behavior.**

Five themes were synthesized out of the in-depth interviews to explain the circumstances in which the bite events happened. Immediate actions taken by bite victims were categorized into two. For those who applied different substances to the bite wounds, the reasons for their choice and actions were recorded into five categories. The same was done to explain why victims went to herbalists, healthcare facilities or had a simultaneous resort.

| **Category** | **Theme** |
| --- | --- |
| (a) Circumstances of the dog bite event | 1) Routine activities bringing dogs and humans into close proximity; 2) Disturbing dogs and threatening their owners; 3) Unusual aggressive behavior and protective tendencies; 4) Deviant dog handling practices and methods; 5) Seasons |
| (b) Immediate actions taken for and by dog bite victims | 1) reporting to authorities and veterinarians; 2) seeking medical care. |
| (c) Explanations for application of non-recommended materials | 1) To kill and remove rabies-virus; 2) Routine management of wounds; 3) Knowledgeable caretakers and trust in herbalist; 4) Pedigree of herbalist; 5) Perceived high cost of conventional treatment; 6) Conflicting information on efficacy of both herbs and modern treatment. |
| (d) Explanations for seeking medical care from hospital | 1) Mistrust in herbalists; 2) Knowledge and experiences on dangers of dog bites; 3) Community advice. |
