# Supplementary material for: Epidemiology and preclinical management of dog bites among humans in Wakiso and Kampala districts, Uganda: Implications for prevention of dog bites and rabies
Source: PLoS One. 2020 Sep 21;15(9):e0239090. doi: 10.1371/journal.pone.0239090 (PMC7505423; doi:10.1371/journal.pone.0239090)
Supplement: S1 File — (PDF) [file pone.0239090.s004.pdf]

APPENDIX IV:
INTERVIEW QUESTIONNAIRE ON FACTORS ASSOCIATED WITH PRE-CLINICAL CARE PRACTICES UNDERTAKEN BY DOG BITE PATIENTS

Appendix IVa: English Version

Date (DD/MM/YY) .... Time respondent had reported to hospital:.................

A. RESEARCH SITE / HOSPITAL NAME : ....

Hospital code: ....

B. RESPONDENT DETAILS

Name code of patient: ....

Residence codes: Village :..Sub county :..

Landmark to home (please describe) : ....

Contact details: Telephone 1 :..Telephone 2: ....

Name of next of kin: Surname :..

Last name :..

Contact details of next of kin : Telephone 1 :..

Telephone 2 :..

Date of birth: Can tell [ ] Cannot remember [ ] Prefers not to say [ ]

If can tell : Date of birth (DD/MM/YY) : ....

If exact date of birth cannot be recalled, what month and year were you born?

(MM/YY)...

C. HUMAN FACTORS

Sex : Male [ ]   Female [ ]

Tribe : ....

Religion : Christian [ ] Moslem [ ] Other .... (please specify)

Highest education level attained : No formal education [ ] Primary [ ] Secondary [ ]

Certificate / Diploma [ ] Degree and above [ ]

Marital status : Single never married [ ] Single divorced / widowed [ ] Married [ ]
Prefer not to say [ ]

Height of respondent (cm) :...

Weight of respondent (kg) :...

How many people stay in the household with you?...

Do the people staying in your household include your spouse? Yes [ ] No [ ]

Are there teenage children in your home? Yes [ ] No [ ]

If yes, how many teenage children in your home? ....

Are you employed? Yes [ ] No [ ]

If yes, please specify type of employment ....

What is your caretaker's sex? Male [ ] Female [ ]

What is your caretaker's education level? No formal education [ ] Primary [ ] Secondary [ ]

Certificate / Diploma [ ] Degree and above [ ]

Are you a Current dog owner? Yes [ ] No [ ]

If yes, how many dogs do you own ? ....

If yes, what type / breed of dog?...

What do you use the dog for? ....

How many years have you been owning the dog (s)?...

If no, have you ever owned a dog? Yes [ ] No [ ]

If yes, where do the dogs stay? Have own house [ ] Out in the compound [ ]

Share house with people [ ] Roam around in the village [ ]

Do you know of any close relative of yours who own a dog? Yes [ ] No [ ]

Have you ever been bitten by a dog before this bite? Yes [ ] No [ ]

If yes, in which year did this bite happen ? ....

For this current bite, did you believe before that a dog could attack and bite you ? Yes [ ] No [ ]

Were you immunized against rabies prior to being bitten by the dog? Yes [ ] No [ ]

If yes, when were you immunized (month and year)? ....

D. DOG FACTORS

What was the sex of the dog? Male [ ] Female [ ] Don't know [ ]

What is the age of the biting dog (months) ....

Was the biting dog sick? Yes [ ] No [ ]

Was the biting dog exhibiting fear of people? Yes [ ] No [ ]

What is the dog used for? Security [ ] Pet [ ] Stray [ ] Dont know [ ]

Was the dog vaccinated to the best of your knowledge? Yes [ ] No [ ] Dont know [ ]

Was the dog spayed / neutered / castrated? Yes [ ] No [ ] Don't know [ ]

Was the dog on the leash? Yes [ ] No [ ]

Has this dog ever bitten someone else before biting you? Yes [ ] No [ ] Don't know [ ]

Did this dog bite another person after biting you? Yes [ ] No [ ] Don't know [ ]

E. DOG BITE CIRCUMSTANCES

E1: Before the bite

What day did the dog bite you? (DD/MM/YY) ....

What time of day did the dog bite you? Morning [ ] Evening [ ] Night [ ]

At what particular time did the dog bite you ? (12-hour format) ....

What it raining when you were attacked by the dog ? Yes [ ] No [ ]

If the attack happended at night, was there a visible moon ? Yes [ ] No [ ]

Were you bitten by your own dog? Yes [ ] No [ ]

If yes, how long had you stayed with the dog before the bite (months) ....

If yes, was this dog borne in your home ? Yes [ ] No [ ]

If yes, do you usually allow this dog into the presence of visitors ? Yes [ ] No [ ]

Does this dog usually leave the compound unaccompanied? Yes [ ] No [ ]

If no, do you know the owner of the dog? Yes [ ] No [ ]

If yes, who is the owner ? Neighbor [ ] Person known to me [ ] Community dog [ ]

Were you bitten while on the property of the dog owner ? Yes [ ] No [ ]

Was the owner around while the dog was attacking you? Yes [ ] No [ ]

How would you describe the size of the dog? Small [ ] Medium [ ] Large [ ] Very large [ ]

Do you know the breed of the dog ? Yes [ ] No [ ]

If yes, what breed was the dog ? ....

Did you previously know the biting dog? Yes [ ] No [ ]

If yes, can you describe the dog? History and type...

Did the dog look sick to you? Ye [ ] No [ ]

Where were you?

Own home [ ] Home of another person known to me [ ] Home of another person not known to me [ ] On the road [ ] Other [ ] , please specify ....

Were you in company of another person / people? Yes [ ] No [ ]

If yes, what is your relationship with these / this person (s)? ....

What were you doing just before the dog bit you? Walking [ ] Seated [ ] Chasing it away [ ] Feeding it [ ] Other [ ] , please specify ....

What was the dog doing just before the bite? Please describe ....

....

Did you try to interpret the mood of the dog before the attack? Yes [ ] No [ ]

Could you describe to me how you thought the dog's demeanor / mood was just before the bite?

....

E2. During the bite

Did you approach the dog or did it approach you? I approached it [ ] It approached me [ ]

Was the dog stationary [ ] or moving / mobile [ ]

What was the purpose of the interaction with the dog around the time of the bite? ....

....

Did you try to fend off the dog as it attacked you? Yes [ ] No [ ]

Where did it bite you? Leg [ ] Hand [ ] Arm [ ] Head [ ] Abdomen [ ]

Other, please specify ....

How many times did it bite you? One [ ] Two [ ] Three or more [ ]

How do you describe the depth of the wounds? Deep [ ] Medium [ ] Superficial [ ]

Other [ ] , please specify ....

Why do you think that the dog bit you? ....

What makes you think that? ....

Do you get information about dogs? Yes [ ] No [ ]

Where do you get information about dogs from? Friends [ ] Books [ ] School [ ] Family [ ]

Other [ ] , please specify..

Do you think that the bite was intentional? Yes [ ] No [ ]

Do you think that anybody is to blame for the bite happening? Yes [ ] No [ ]

If yes, who? ....

E3. After the bite

How would you describe the damage the bite did to you? ....

What did you do to the dog after the bite? Chased it away [ ] Killed it [ ] Nothing [ ]

It ran away by itself [ ] Other [ ], please specify ....

If it was killed, what happened to the carcass ? Decapitated [ ] Buried [ ] Left to rot [ ]

I don't know [ ] Other [ ] , please specify ....

Are you aware the head of the dog had to be taken for examination ? Yes [ ] No [ ]

If yes, was the head take for examination? Yes [ ] No [ ]

If not own dog, how did the owner react? ....

How did the owner's reaction make you feel? ....

How did the bite affect the rest of your day? ....

F. PRACTICES

Did you do anything to the wound immediately after the bite? Yes [ ] No [ ]

If yes, what did you do? Washed with water and soap [ ] Washed with water only [ ]

Did not wash [ ] Other, please specify ....

Did you apply anything to the wound immediately after the bite? Yes [ ] No [ ]

If yes, what did you apply to the wound? ....

Did you think you needed any medical help after the bite? Yes [ ] No [ ]

If yes, what did you do? ....

Why did you choose to do that? ....

G. SES variables

Could you tell me if you have the following in your house;

Item	Yes	No
Radio		
Television		
Cell-phone		
Bicycle		
Motorcyle		
Motor vehicle		
A piece of land		
Large farm animals like cattle, goats and sheep		
Small farm animals like poultry		
A manufactured bed		

What is the nature of the walls of their house?

No bricks [ ] Unburnt bricks [ ] Burnt bricks with mud [ ]

Burnt bricks/stones with cemen [ ] Other [ ] , please specify ....
