# Supplementary material for: Epidemiology and preclinical management of dog bites among humans in Wakiso and Kampala districts, Uganda: Implications for prevention of dog bites and rabies
Source: PLoS One. 2020 Sep 21;15(9):e0239090. doi: 10.1371/journal.pone.0239090 (PMC7505423; doi:10.1371/journal.pone.0239090)
Supplement: S2 File — (PDF) [file pone.0239090.s005.pdf]

# APPENDIX IVB:
## LUGANDA VERSION

### EBIBUUZO EBIKWATA KU NSONGA EZIVIIRAKO ABALUMIDDWA EMBWA OKUKOLA EBYO BYE BAKOLA NGA TEBANAGENDA MU DDWALIRO

Ennaku z'omwezi leero (Olunaku/Omwezi/Omwaka) ........................................................................................................

Obudde omulwadde lwazze mu ddwaliro:........................................................................................................

**A. Erinnya ly'eddwaliro:**........................................................................................................

Namba y'eddwaliro: ........................................................................................................

**B. Ebikwata ku mulwadde**

Amannya (mu bwekusifu) : ........................................................................................................

**Ekifo mwobeera (mu bwekusifu)** : Ekyaalo........................................................................................................

Eggombolola:........................................................................................................

Ekiraga wobeera (nyonyola): ........................................................................

**Essimu:** Esooka ........................................................................................................

Endala........................................................................................................

**Eyebuzibwaako nga muntu wa mulwadde:**

Erinnya ly'ekika ........................................................................................................

Ezzungu........................................................................................................

**Essimu z'oyo eyebuzibwaako :** Esooka:........................................................................................................

Endala........................................................................................................

**Wazaliibwa ddi :** Nzijukira [ ] Sijukira [ ] Ssandyagadde kukubulira [ ]

**Bwooba ojjukira :** Amazaliibwa (Olunaku/Omwezi/Omwaka): ........................................................................

........................................................................................................

Bwooba tojjukira lunaku lwe wazaliibwa, mwezi ki era mwaka ki lwewazaalibwa? (Mwezi/Mwaka)

....................................................................................................................................................................

**C. Ensonga ez'obuntu**

**Enkula :** Musajja [ ]  Mukyala [ ]

Eggwanga :.......................................................................................................................................................

**Eddiini :** Mukurisitaayo [ ] Musiraamu [ ] Ekirala .............................................................. (laga ekirala)

**Obuyigirize obusembayo waggulu :** Teyasoma [ ] Pulayimale [ ] Sekendule [ ] Satifukeeti / Dipulooma [ ] Diguli n'okweyongerayo [ ]

**Obufumbo :** Tafumbirangaako [ ] Simufumbo naye yayawukana oba yafiirwa munne [ ] Mufumbo [ ] Teyandyagadde kutubulira [ ]

Obuwanvu (cm)....................................................................Obuzito (kg) ...................................................................

Obungi bwa bantu bobeera nabo ewaka .........................................................................................................

**Bobeera nabo kuliko omubeezi?** Yee [ ] Nedda [ ]

**Ewaka wobeera waliwo abaana abatiini?** Yee [ ] Nedda [ ]

Bwewaba ewaka waliwo abatiini, bali bameka? .........................................................................................

**Olina omulimu?** Yee [ ] Nedda [ ] Bwooba olina omulim, tukusaba ogutugambe...............................................

**Akulabirira mu ki?** Musajja [ ]  Mukazi [ ]

**Akulabirira wabuyigirize ki?** Teyasoma [ ] Pulayimale [ ] Sekendule [ ] Satifikeeti / Diplooma [ ] Diguli n'okwambuka [ ]

**Olina embwa gy'okuuma?** Yee [ ] Nedda [ ]

Oba olina embwa, ziri mmeka? ...........................................................................................................................

Oba olina embwa, ya kika ki (nganda oba nzungu)? ................................................................................

Embwa gyolina ya mugaso ki? ...........................................................................................................................

Embwa ogikumidde / oziikumidde bbanga ki? ..............................................................................................

**Bwooba tolina mbwa kati, wali obadde nayo?** Yee [ ] Nedda [ ]

**Bwooba olina embwa, zibeera wa?** Mu nyumba yaazo [ ] Mu luggya [ ] Zisula naffe mu nyumba [ ] Zitayaaya ku kyaalo [ ]

**Waliyo ow'oluganda lwo gw'omanyi nga alina embwa?** Yee [ ] Nedda [ ]

**Wali olumiddwaako ebwa ebbanga eriyise nga eno tenakuluma?** Yee [ ] Nedda [ ]

Oba wai olumiddwaako, kyaliwo mwaka ki? ..............................................................................................

**Luno oluluma olubaddewo kati: wali okisubiira nti embwa esobola okukuluma?** Yee [ ] Nedda [ ]

**Wali ogemeddwa rabies / obulwadde bw'embwa obw'eddalu nga embwa tenakuluma?**

Yee [ ] Nedda [ ]

Oba wali ogemeddwa, gwali mwezi ki era mwaka ki? ..............................................................................

## D. ENSONGA EZEKUUSA KU MBWA EYAKULUMYE

**Obutonde bw'embwa?** Nsajja [ ] Nkazi [ ] Simanyi [ ]

Embwa eno ya bukulu ki (mu myeezi, bwooba omanyi) ..............................................................................

**Embwa yabadde ndwadde?** Yee [ ] Nedda [ ] Simanyi [ ]

**Embwa yabadde eraga nti etya abantu?** Yee [ ] Nedda [ ]

**Embwa eno yamugaso ki?** Kukuuma waka [ ] Yakuzanyisa [ ] Etayaaya [ ] Simanyi [ ]

**Mukumanya kwo, embwa eno baali bagigema?** Yee [ ] Nedda [ ] Simanyi [ ]

**Embwa eno baali bagigema okuzaala (okulaawa)?** Yee [ ] Nedda [ ] Simanyi [ ]

**Embwa eno yaki kulujegere oba kumuguwa?** Yee [ ] Nedda [ ]

**Embwa eno yali erumye ku muntu omulala nga tenakuluma?** Yee [ ] Nedda [ ] Simanyi [ ]

**Embwa eno yalumye omuntu omulala nga emaze okukuluma?** Yee [ ] Neddao [ ] Simanyi [ ]

## E. ENSONGA EZETOLOORERA KU KULUMWA KO

### E1: Nga embwa tenakuluma

**Embwa yakuluma lunaku ki?** (Olunaku/Omwezi/Omwaka)........................................................................

**Embwa yakuluma ssaawa meka?** Ku makya [ ] Lwa ggulo [ ] Kiro [ ]

**Embwa yakuluma ssaawa mekka ddala?** (12-hour format) ........................................................................

**Embwa yakuluma enkuba ettonya?** Yee [ ] Nedda [ ]

**Embwa bweeba yakuluma kiro, waaliyo omwezi waggulu mu bwengula?** Yee [ ] Nedda [ ]

**Embwa eyakuluma, yiyo?** Yee [ ] Nedda [ ]

Oba yiyo, wali wakamala nayo bbanga ki (myeezi)? ........................................................................

**Oba yiyi, yazalibwa waka wo?** Yee [ ] Nedda [ ]

**Oba yiyo, otera okugita nga waliwo abagenyi?** Yee [ ] Nedda [ ]

**Embwa eno etera okuva ewaka nga teri na muntu?** Yee [ ] Nedda [ ]

**Oba embwa eno siyiyo, omanyi nyini yo?** Yee [ ] Nedda [ ]

**Bwooba omanyi yini yo, yaani?** Mulirwana [ ] Twemanyi [ ] Yakukyaalo [ ]

**Embwa yakulumidde wa nyini yo?** Yee [ ] Nedda [ ]

**Embwa yakulumye nga nyini yo waali?** Yee [ ] Nedda [ ]

**Obunene bw'embwa eno obunyonyola otya?** Ntono [ ] Yakitema [ ] Nene [ ] Nene nyo [ ]

**Omanyi ekikula ky'embwa eno?** Yee [ ] Nedda [ ]

Oba omanyi ekikula kyaayo (breed), kitubuliire ........................................................................

**Embwa eno wali ogimanyi nga tenakuluma?** Yee [ ] Nedda [ ]

Bwooba wali ogimanyi, tunyonyole ebogikatako ........................................................................

**Embwa eno yakulabikidde ng'endwadde?** Yee [ ] Nedda [ ]

**Embwa yakulumidde wa?** Ewaffe [ ] Awaka w'omuntu omulala gwemanyi [ ] Awaka w'omuntu

omulala gwe simanyio [ ] Mu luguudo [ ] Walala [ ] , tubuliire ewalala ..........................................

**Yakulumye li n'omuntu omulala oba abantu abalala?** Yee [ ] Nedda [ ]

Oba wabadde n'omuntu omulala, omuyita otya? ..........................................................................................

**Wali okola ki nga embwa tenakuluma?** Nga ntambula [ ] Nga ntudde [ ] Nga ngigoba [ ]

Nga ngiriisa [ ] Ekirala [ ] , tubuliire ekirala ..........................................................................................

**Embwa yabadde ekola ki nga tenakuluma?** Nyonyola...........................................................................................

...................................................................................................................................................................

**Wagezaako okusoma embbera y'embwa (oba nyiivu) nga tenaba kukwangaanga?**

Yee [ ] Nedda [ ]

Tubuliire embeera y'embwa eno gyayabaddemu nga tenakulumba ...............................................................

...................................................................................................................................................................

**E2. Mu kiseera nga embwa ekuluma**

**Gwe wasemberede embwa oba yeyakusemberedde?**

Nze nagisemberedde [ ] Yeyansemberedde [ ]

Embwa yabadde mu kifo kimu oba nga etambula? ..........................................................................................

Okwetaba n'embwa eno kyabadde kigendererwaki? ..........................................................................................

**Wagezezaako okwerwanako nga embwa ekuluma?** Yee [ ] Nedda [ ]

**Yakulumye wa?** Kugulu [ ] Kibatu [ ] Mukono [ ] Mutwe [ ] Lubuto [ ]

Walala, tubuliire ewalala ..........................................................................................................................

**Embwa yakulumye emirundi emeka?** Gumu [ ] Ebiri [ ] Esatu n'okusoba [ ]

**Ekiwundu okuyingira munda okinyonyola otya?**

Kiyingidde nnyo [ ] kyakitema [ ] Kiri kungulu [ ]

Olowooza lwaki embwa yakulumye? ..............................................................................................................

Lwaki olowooza bwootyo? ..............................................................................................................

**Ofuna obubaka bwonna obwekuusa ku mbwa?** Yee [ ] Nedda [ ]

**Obubaka obukwata ku mbwa obujja wa?** Mikwano [ ] Bitabo [ ] Ssomero [ ] Ob'oluganda [ ]

Walala [ ] nyonyola ewalala ..............................................................................................................

**Olowooza okukuluma kyabadde kigenderere?** Yee [ ] Nedda [ ]

**Olowooza waliwo omuntu yenna owokuvunaana ku kulumwa kwo?** Yee [ ] Nedda [ ]

Oba waali, y'ani? ..............................................................................................................

**E3. Nga embwa emaze okukuluma**

Ekiwundi kino oba ebiwundu bino obyogerako otya? ..............................................................................................................

**Wakoze ki nga embwa emaze okukuluma?** Nagigobye [ ] Yatiddwa [ ] Tewali [ ] Yadduse [ ]

Ekirala, kinyonyole ..............................................................................................................

**Bweeba yatiddwa, omutulumbi gwabadde ki?** Bagitemyeeko omutwe [ ] Yazikiddwa [ ]

Yalekeddwa awo [ ] Simanyi [ ] Ekirala, nyonyola ..............................................................................................................

**Okimanyi nti omutwe gw'embwa gulina okutalibwa okwekebejjebwa?** Yee [ ] Nedda [ ]

**Bwooba okimanyi, omutwe gwatwaliddwa okugwekebejja?** Yee [ ] Nedda [ ]

Bweeba nti embwa teyabadde yiyo, nyiniyo yakozeewo ki?..............................................................................................................

Wawulidde otya ku kikolwa kya nyini yo? ..............................................................................................................

Ebiwundi byakosezza bitya olunaku lwo? ..............................................................................................................

**F. EBYAKOLEDDWA NGA EMBWA EMAZE OKUKULUM**

**Olina kywakoze ku kiwundu nga embwa emaze okukuluma?** Yee [ ] Nedda [ ]

**Oba yee, wakoze ki?** Nayozezza ekiwundu ne ssabuni nga ali mu mazzi [ ]

Nayozezza n'amazzi gokka [ ] Sayozezza [ ] Ekirala, nyonyola ............................................................

**Olina kyawatadde ku kiwundu nga embwa emaze okukuluma?** Yee [ ] Nedda [ ]

Oba yee, wataddeko ki? ..........................................................................................................................

**Walowozezza nti wetaaga obujanjabi nga embwa emaze okukuluma?** Yee [ ] Nedda [ ]

Oba yee, wakoze ki? ................................................................................................................................

Lwaki wasazeewo okukola ekyo? ............................................................................................................

## G. EBIKWAATA KU BUGAGGA BWO

Mbulira oba ewaka wo waliyo ebintu bino wammanga;

| EKINTU | YEE | NEDDA |
|---|---|---|
| Leediyo | | |
| Tivvi | | |
| Akasimu | | |
| Akagaali | | |
| Pikipiki | | |
| Motoka | | |
| Ettaka | | |
| Ebisolo ebinene nga ente, embuzi, endiga | | |
| Ebisoo ebitono nga enkoko | | |
| Ekitanda ekibajje | | |

**Ebisenge by'enyumba byazimbibwa naki?**

Si na bulloka [ ] Bulooka ezitali njokye [ ] Bulooka enjokye n'akadongo [ ]

Bulooka enjokye ne sementi [ ] Ekirala [ ] , nyonyola ..................................................................................
