# Supplementary material for: Epidemiology and preclinical management of dog bites among humans in Wakiso and Kampala districts, Uganda: Implications for prevention of dog bites and rabies
Source: PLoS One. 2020 Sep 21;15(9):e0239090. doi: 10.1371/journal.pone.0239090 (PMC7505423; doi:10.1371/journal.pone.0239090)
Supplement: S3 File — (PDF) [file pone.0239090.s006.pdf]

In-depth interview guide on pre-clinical care practices undertaken by dog bite patients (for the patients)

Title of study: PRE-CLINICAL CARE, CLINICAL MANAGEMENT AND OUTCOMES OF DOG BITE INJURIES IN HIGH RABIES BURDEN DISTRICTS OF WAKISO AND KAMPALA, UGANDA

Date: --- Time: --

Interviewee ID: ---

"Good morning / good afternoon / good evening. I am ________ (introduce self).

This interview is being conducted to get to deeply understand what you did after the dog bit you but before presenting to the health facility. I am especially interested in what motivated you to manage the dog bite wound the way you did.

If it is okay with you, I will be tape recording our conversation. The purpose of this is so that I can get all the details but at the same time be able to carry on an attentive conversation with you. I assure you that all your comments will remain confidential. I will be compiling a report which will contain all patient comments without any reference to individuals. If you agree to this interview and the tape recording, there is no need to sign another consent form since all was explained to you at the beginning.

Ground rules

Before we start I would like to remind you that there are no right or wrong answers in this discussion. We are interested in knowing what you think, so please feel free to be frank and to share your point of view. It is very important that we hear your opinion.

1. Why do you think the dog bit you?
2. What could you have done to prevent the dog from biting you?
3. What threatens you about this dog bite injury?
4. How has the bite affected your life?
5. What did you do to the wound after the dog bit you? Please explain.
6. Why did you do what you did? Please explain.
7. Could you please explain anything you know about infection of dog bite wounds?
8. Who decided that you come to the hospital to receive treatment?
9. Do you know of any dangers that may accrue from not reporting the injuries to hospital?
10. Why did it take you so long to report to health facility?
11. Anything you know about rabies? Probe for transmission, prevention and consequences.
12. Why were you not vaccinated against rabies? Please explain.

13. Are you planning to do anything in the future to prevent another bite from happening? Please explain.
14. Let’s summarize some of the key points from our discussion. Is there anything else?
15. Do you have any questions?

Thank you for taking the time to talk to me!!

Key informant interview guide on pre-clinical care practices undertaken by dog bite patients (for healthworkers)

Title of study: PRE-CLINICAL CARE, CLINICAL MANAGEMENT AND OUTCOMES OF DOG BITE INJURIES IN HIGH RABIES BURDEN DISTRICTS OF WAKISO AND KAMPALA, UGANDA

Date: --- Time: --

Interviewee ID: --

My name is **_________** from University of Nairobi Institute of Tropical and Infectious Diseases and Makerere University School of Public Health. I am working on a research that is trying to establish the practices of dog bite victims before they come to report to the health facility. The findings of this research may be used to create awareness of what people have to do in case they are bitten by dogs. They may also be used to improve the treatment and care that such patients receive when they come for medical attention. This is because an important step in caring for dog bite victims is establishing whether they followed the recommended guidelines of irrigating the wound with water and soap before seeking medical care. The themes that emerge from the interview process will inform our planned recommendations. The key informant interview itself will be confidential. Nothing you say will be personally attributed to you in any reports that result from this interview. All of our reports will be written in a manner that no individual comment can be attributed to a particular person. Your knowledge will be very valuable and the interview will last only 20 minutes if you consent. Do you have any questions or concerns before we begin?

1. What challenges do you think the dog bite victims face in regard to management of the bite wound?
2. In what ways do dog bite victims drift away from guidelines of dog bite wound management before seeking medical care?
3. From your own perspective, why do you think some dog bite victims do not adhere to the guidelines?
4. In what ways does not adhering to the guidelines affect the patients?
5. If the patient does not adhere to these guidelines before seeking medical care, how does it affect your work as a health worker?
6. How have you tried to ensure that the dog bite victims adhere to pre-clinical guidelines?

7. Do you have any additional comments about the pre-clinical care for dog bite victims that we haven't already discussed?

Thank you for taking the time to talk to us!!

Key informant interview guide on pre-clinical care practices undertaken by dog bite patients (for veterinarians)

Title of study: PRE-CLINICAL CARE, CLINICAL MANAGEMENT AND OUTCOMES OF DOG BITE INJURIES IN HIGH RABIES BURDEN DISTRICTS OF WAKISO AND KAMPALA, UGANDA

Date: --- Time: ---

Interviewee ID: --

My name is **_________** from University of Nairobi Institute of Tropical and Infectious Diseases and Makerere University School of Public Health. I am working on a research that is trying to establish the practices of dog bite victims before they come to report to the health facility. The findings of this research may be used to create awareness of what people have to do in case they are bitten by dogs. They may also be used to improve the treatment and care that such patients receive when they come for medical attention. This is because an important step in caring for dog bite victims is establishing whether they followed the recommended guidelines of irrigating the wound with water and soap before seeking medical care. The themes that emerge from the interview process will inform our planned recommendations. The key informant interview itself will be confidential. Nothing you say will be personally attributed to you in any reports that result from this interview. All of our reports will be written in a manner that no individual comment can be attributed to a particular person. Your knowledge will be very valuable and the interview will last only 20 minutes if you consent. Do you have any questions or concerns before we begin?

1. Tell me about your involvement and role in the community when it comes to dog bites.
    - Activities to prevent dog bites
    - Roles of the veterinarian when a person is bitten
2. From your knowledge and experience in the community, what do people do after being bitten by a dog? Investigate for;
    - Compliance to pre-clinical guidelines by Ministry of Health
    - Reporting mechanisms in place
    - Practices to try to treat the wounds
3. Why do you think the victims do such things? (ask on the specific practices individually)
4. What challenges do you think the dog bite victims face in regard to management of the bite wound?
5. From your own perspective, why do you think some dog bite victims do not adhere to the guidelines?
6. In what ways does not adhering to the guidelines affect the patients?

7. If the patient does not adhere to these guidelines before seeking medical care, how does it affect your work as a veterinarian?
8. How have you tried to ensure that the dog bite victims adhere to pre-clinical guidelines?
9. Do you have any additional comments about the pre-clinical care for dog bite victims that we haven't already discussed?

Thank you for taking the time to talk to us!!

Key informant interview guide on pre-clinical care practices undertaken by dog bite patients (Traditional healers)

Omulamwa gw'okunonyereza: EBIKOLEBWA NGA EMBWA ERUMYE OMUNTU, OBUJANJABI OBUGABIRWA MU DDWALIRO NE BIKI EBIVA MUKUJANJABA EBIWUNDU EBIRETEBWA EMBWA MU WAKISO NE KAMPALA DISITULIIKITI MU YUGANDA

Ennaky z'omwezi: --Obudde: -------------------------

Addamu ebibuuzo: ---

Errinya nze _________ okuva mu Yunivasite y'e Nairobi e Kenya n'e Yunivasite y'e Makerere mu ssomero ly'ebyobulamu eby'olukale. Ndi mu kunonyereza ku biki abantu embwa bezirumye bywbakola nga tebanagenda mu malwaliro gano amazungu. Ebinaava mu kunonyereza bisobola okukozesebwa okwongera okubangula abantu mu kiki kyebalina okukola nga embwa zibalumye. Bisobola no kukozesebwa okusitula omutindo gwe ngeri gyebajanjabwamu. Byonatubulira byonna bijja kukumibwa nga bya kyaama eri teri binavaamu bijja kutekebwaako kakwaate ku ggwe. Alipoota yaffe tujja kugiwandiika mu ngeri etalaga nti ggwe wayogera ebyo. By'omanyi bya muwendo gyetuli era okubuuza kwaffe kujja kutwaala eddakiika nga abiri bwoba okkirizza okwetaba mu kubuliriza kuno. Olina kye wandyagadde okubuuza nga tetunatandika?

1. Abalwadde bojjanjaba bava wa?
    - Ebyalo ne district
2. Mw'abo b'ojanjaba mulimu embwa beziba zirumye. Obakolera ki nga bazze ewuwo?
3. Lwaki okola ebintu ebyo ku bantu embwa bezirumye?
4. Bagenda okujja baba basoose kukola ki?
5. Waliwo abakomawo ne bakugamba nti bawonye? Olabira ku ki nti bawonye?
6. Lwaki abantu bajja ewuwo ne batagenda mu ddwaliro?
7. Olowooza lwaki embwa ziruma abantu?
8. Waliwo obulabe bwonna bwomanyi obuva ku mbwa okuluma omuntu? Lwaki olowooza nti buno bulabe?
9. Wandyagadde kiki ekiba kikolebwa okutangira embwa okuluma abantu?
10. Olina byosobola okwongera ku mboozi yaffe eno by'olowooza nti tetubikutteeko?

Tukwebaza okutuwa obudde okwogerako naffe!!
